# Amelioration of Cadmium-Induced Nephropathy using Polyphenol-rich Extract of *Vernonia amygdalina* (Del.) Leaves in Rat Model

**DOI:** 10.3889/oamjms.2015.120

**Published:** 2015-11-27

**Authors:** Christian E. Imafidon, Rufus O. Akomolafe, Sanusi A. Abubakar, Oluwadare J. Ogundipe, Olaoluwa S. Olukiran, Oladele A. Ayowole

**Affiliations:** 1 *Obafemi Awolowo University, Department of Physiological Sciences, Ile-Ife, Osun, Nigeria*; 2 *Obafemi Awolowo University, Department of Medicine, Ile-Ife, Osun, Nigeria*; 3 *Afe Babalola University - Department of Medical Laboratory Science, College of Medicine, Ado Ekiti, Ekiti, Nigeria*

**Keywords:** Cadmium, Polyphenol - Rich Extract of leaves of *Vernonia amygdalina* (PEVA), Nephrotoxicity, Creatinine clearance, Ameliorative, Rats

## Abstract

**AIM:**

To determine the effects of polyphenol-rich extract of the leaves of *Vernonia amygdalina* (PEVA) in rats with Cd-induced nephropathy.

**MATERIALS AND METHODS:**

Sixty five male Wistar rats were divided into five groups as follows; Group 1 received distilled water throughout the period of study. Group 2 received 5 mg/kg body weight of cadmium (Cd), in the form of CdSO_4_, for five consecutive days via intraperitoneal route. Groups 3, 4 and 5 were pretreated with Cd as group 2 and thereafter received oral treatment of PEVA for 4 weeks at 100 mg/kg, 200 mg/kg and 400 mg/kg body weight, respectively.

**RESULTS:**

Exposure to Cd toxicity significantly induced deleterious alterations in plasma and urine levels of creatinine, urea and glucose as well as creatinine and urea clearance (p < 0.05) in the rat model. There was a significant disturbance in the antioxidant system as revealed by the levels of thiobarbituric acid reactive substance (TBARS) and reduced glutathione (GSH) (p < 0.05) in the kidney tissue of the rats. With marked improvements in renal histoarchitecture, PEVA treatment showed a duration and non dose-dependent ameliorative potential.

**CONCLUSION:**

PEVA treatment reversed the compromise of renal function that was induced by Cd toxicity in rat model.

## Introduction

Cadmium is a ubiquitous non-essential heavy metal of importance in several industrial processes [1]. Its inability to undergo metabolic degradation to less toxic metabolites makes its exposure a serious health concern [2]. There is increasing concern about its contamination of soil and water as it bioaccumulate in the food chain, particularly in regions of inadequate exposure-control. Hence, consumption of contaminated food is a greater part of its intake in humans [3, 4]. A further contribution to human exposure is the considerable portion it occupies in tobacco/cigarette [1, 4], thus exerting its toxic effects in both active and passive smokers. Unlike most heavy metals, exposures to cadmium can induce disruptions in a number of biological activities and systems at relatively lower doses [5-7]. Its toxicity, basically, affects the vital organs of the body. However, its main repository organ is the kidney [4, 8, 9].

According to the National Association of Nephrology (NAN, Nigeria), about 30 million Nigerians have chronic kidney disease [10]. This staggering figure was corroborated by the National Kidney Foundation (NFK, USA) that about 27 million Nigerians have chronic kidney disease. This precursor to kidney failure is with an incidence of 100 per million populations (about 15,000 new cases every year) and prevalence of 300 to 400 million (about 45,000 living with kidney failure annually) [10]. The incidence of kidney disease is on the increase (worldwide) with high cost of management or treatment. Cadmium is reputed for its nephrotoxic effect. The use of established antagonists and various chelating agents to reduce its toxicity is usually associated with undesirable side effects [11]; hence current scientific research is geared towards exploiting the health benefits of natural products of plant origin. These plants are usually common and relatively cheaper, yet with promising health-boosting potentials.

Various vegetables, fruits and whole grains are examples of abundant sources of polyphenols [12]. Polyphenols are strong antioxidants which act as a defense against reactive oxygen species (ROS)-induced oxidative stress [13]. They are reputed for complementing and adding to the functions of antioxidant enzymes and vitamins [13]. The study of the biological effects of polyphenols has become an area of interest in the light of recent advances in the field of nutrition and medical sciences [14].

*Vernonia amygdalina* leaf (commonly called bitter leaf) is reputed for its many medicinal benefits which range from anti-inflammatory, antidiabetic [10], bad and total cholesterol-reducing [15], cardiovascular disease-ameliorating [16], immune system-strengthening, anti-microbial [10], anti-diarrhea, and anti-emetic [17] potentials, to mention a few. However, the health benefits of this apparently medicinal plant have been attributed, by most literature, to the presence of polyphenols in its extract [15-17]. The effects of this polyphenol-rich extract on cadmium-induced kidney injury have not been reported, hence this study.

## Material and Methods

### Plant, Materials and Chemicals

Fresh leaves of *Vernonia amygdalina* were obtained from a garden in Ife-Ibadan area of Ile-Ife, Osun State, Nigeria. This was certified by a Taxonomist (Mr. A. Gabriel) in the Department of Botany, Obafemi Awolowo University (OAU), Ile-Ife, Osun State, Nigeria.

Ohaus R Model of metabolic cage (Ohaus, Pine Brook, New Jersey, USA) was used for this study. Standard Laboratory kits for assay of indices of renal function were purchased from Randox Laboratories Limited, United Kingdom. Acetone was purchased from Crescent Chemical Co., Inc, New York, United States while Cadmium sulphate was purchased from Guangzhou Fischer Chemical Co., Ltd, Guangdong, China.

### Extraction of Polyphenols

Using standard procedure, polyphenol-rich extract of leaves of *Vernonia amygdalina* (PEVA) was obtained as follows; the leaves were air-dried and pulverized with an Electric Pulverizer (DIK-2910, Daiki Rika Kogyo Co. Ltd, Tokyo-Japan) and thereafter weighed. This was further crushed in 80% acetone (1:2 w/v) using a Waring blender (Waring Commercial, Torrington, CT). The sample was homogenized in a Polytron Homogenizer (Glen Mills Inc., Clifton, NJ) for 3 minutes and the homogenates was filtered under vacuum using Buchner funnel and Whatman number 2 Filter Paper (Whatman PLC, Middlesex, UK). The filtrate was concentrated under vacuum using a Rotary Evaporator (HahnShin Scientific, HS-2005-N) and freeze-dried in a Lyophilizer (Ilshin Lab. Co. Ltd, Seoul, Republic of Korea). The yield obtained (PEVA) was weighed and kept in a desiccator until when needed. The percentage (%) yield of PEVA was calculated as shown below;

% yield of PEVA = yield of PEVA ÷ weight of pulverized leaves x 100 % [18].

As reported in our earlier study, the total phenol content was determined to be 681 ± 47.36 mg of gallic acid equivalent per gramme of the extract while the total flavonoids content was 23.70 ± 1.78 mg of quercetin equivalent per gramme of the extract [19].

### Solutions of PEVA Extract and Cadmium Salt

Polyphenol-rich extract of leaves of *Vernonia amygdalina* (PEVA) was reconstituted with distilled water to prepare three stock solutions. One gramme (1 g) of PEVA was dissolved in 20 ml of distilled water to obtain a stock solution for 100 mg/kg of PEVA. Also, 2 g and 4 g of PEVA were each dissolved in 20 ml of distilled water to obtain stock solutions for 200 mg/kg and 400 mg/kg of PEVA, respectively. This implies that the rats received graded doses of PEVA at 100, 200 and 400 mg/kg body weight, respectively. The choice of therapeutic doses for this study was guided by the predetermined oral LD50 of PEVA (and taken to be ≤ 10% of oral LD50), as reported in our previous study [19]. Therefore, the extracts were administered at 0.2 ml per 100 g body weight of rats. Fresh samples were prepared every 48 hours and stored in a deep-freezer after use.

Fifty milligram (50 mg) of cadmium sulphate salt was dissolved in 20 ml of distilled water and administered to rats at 0.2 ml/100 g bodyweight. This implies that 0.5 mg of cadmium solution was administered to 100 g rat. Renal injury was induced by the administration of solution of Cd at a dose of 5 mg/kg/day for five consecutive days, via intraperitoneal route (i.p.).

### Animal Management and Experimental Protocol

The experimental protocols were in strict compliance with the guide for animal research, as detailed in the NIH Guidelines for the Care and Use of Laboratory Animals (National Academy of Sciences and National Institutes of Health Publications, 2011) [20] and approved by local Institutional Research Committee. Sixty five (65) male Wistar rats, weighing 150-175 g, were used for this study. They were purchased from the Animal Holdings of the College of Health Sciences, OAU, Ile-Ife, Osun State, Nigeria; where the study was carried out. Each rat was housed in a separate metabolic cage under natural light/dark cycle and allowed to have access to standard laboratory rat chow (Caps Feed PLC Osogbo, Nigeria) and water *ad libitum*. The rats were allowed to acclimatize in the metabolic cage for 2 weeks before the commencement of this study, to allow for adaptation to life in metabolic cage.

**Table 1:** Experimental Protocol and Dose Regimen

	DAY 5	WEEK 2	WEEK 4	WEEK 6
GROUP 1	DW	DW	DW	DW*
GROUP 2	Cd*	RP*	RP*	
GROUP 3	Cd	P 100 mg/kg*	100 mg/kg*	RP *
GROUP 4	Cd	P 200 mg/kg*	200 mg/kg*	RP *
GROUP 5	Cd	P 400 mg/kg*	400 mg/kg*	RP*

DW= Distilled water; Cd= Cadmium (5 mg/kg bw via intraperitoneal route); P= Polyphenol-rich extract of leaves of *Vernonia amygdalina* (PEVA); RP= Recovery period; Group 1= Control (5 rats); Group 2= Toxic Control (15 rats); Group 3= Cd + 100 mg/kg PEVA (15 rats); Group 4= Cd + 200 mg/kg PEVA (15 rats); Group 5= Cd + 400 mg/kg PEVA (15 rats);

* = point that rats were euthanized.

The rats were divided into five (5) groups as follows; Group 1 (control group) consisted of 5 rats that received distilled water at 0.2 ml/100 g body weight, throughout the study period (6 weeks) after which they were sacrificed. Group 2 (toxic group) consisted of 15 rats each of which received Cadmium (Cd) at 5 mg/kg in the form of CdSO_4_ via intraperitoneal route, once daily, for five consecutive days. Five of the rats were sacrificed 24 hours after Cd intoxication.

From the remaining 10 rats, 5 each were sacrificed after a recovery period of 2 and 4 weeks. Groups 3, 4 and 5 consisted of 15 rats each, which were also intoxicated with Cd as stated above. Thereafter, they received 100, 200 and 400 mg/kg of PEVA respectively, via oral route of which 5 rats were sacrificed after 14 days. The remaining 10 rats in each group received the respective doses of PEVA for 28 days of which 5 rats were sacrificed, twenty four hours, after the last dose of PEVA while the remaining 5 rats were sacrificed after 2 weeks of recovery period.

Blood samples were collected by cardiac puncture into separate EDTA bottles and centrifuged at 4000 rpm for 15 minutes with the aid of a cold centrifuge (Centurium Scientific, Model 8881) at -4ºC in order to obtain the plasma. Plasma obtained was collected into separate plain bottles for the assessment of the indices of renal function.

### Measurement of Body Weight and Food Consumption

With the aid of a digital weighing balance (Hanson, China), weekly body weight and food consumption of the rats were measured. The food consumption was measured by subtracting the final amount from the initial amount that was measured a day before. The value obtained was taken to be the amount consumed by each rat.

### Collection and Measurement of Urine Samples

With the aid of the metabolic cage, urine samples of rats were collected into separate plastic containers. Urine volumes were read off directly with the aid of a measuring cylinder and volumes that were less than 1ml were measured with the aid of an insulin syringe.

### Biochemical Assays

Plasma and urine levels of creatinine, urea and glucose were estimated using standard laboratory protocols, as provided by Randox Laboratories. Creatinine and urea clearance were calculated using conventional formulae as follows;

Clearance= UV/P (ml/min).

Where U= concentration of substance in urine;

V= urine flow rate = amount of urine/time (secs); and

P= concentration of substance in plasma.

### Homogenate Preparation

The kidneys of the rats were carefully excised and weighed. One of the kidneys (left) was homogenized with 10ml of sucrose solution (0.25M) using Electric Homogenizer (SI601001). 10% homogenate in phosphate buffer (100 Mm) was prepared at pH of 7.4. The homogenate was centrifuged at 3000 rpm for 20 minutes and the supernatant was collected for the assessment of levels of lipid peroxidation and oxidative stress.

### Assay for Non-enzymatic Antioxidant Status

Reduced glutathione (GSH) levels were measured by the method of Beutler and Kelly [21]. 1 ml of the supernatant, obtained above, was added to 0.5 ml of Ellman’s reagent (10 mM). 2 ml of phosphate buffer (0.2 M, pH 8.0) was, thereafter, added. The yellow colour developed was read at 412 nm against blank containing 3.5 ml of phosphate buffer. A series of standards were also treated similarly and the amount of GSH was expressed in mg/100 g tissue.

### Assay for Lipid Peroxidation

Levels of thiobarbituric acid reactive substance (TBARS) were determined by the method of Ohkawa *et al* [22]. To each 0.5 ml of the supernatant was added 0.5 ml of phosphate buffer (0.1 M, pH 8.0) and 0.5 ml of 24% TCA. The resulting mixture was incubated at room temperature for 10 min, followed by centrifugation at 2000 rpm for 20 min. To 1 ml of the resulting supernatant was added 0.25 ml of 0.33% TBA in 20% acetic acid and the resulting mixture was boiled at 95°C for 1 hr. The resulting pink colour product was cooled and absorbance was read at 532nm (Extinction coefficient of TBARS; ε532 = 1.53 × 105 M^-1^ cm^-1^).

### Histopathological Studies

The right kidneys of the rats were fixed in 10% formal-saline solution. They were, thereafter, dehydrated in graded alcohol and embedded in paraffin wax. Sections taken (7-8 µm thick) were stained, using Hematoxylin and Eosin (H & E) technique, for photomicroscopic assessment with the aid of Leica DM 750 camera microscope at magnifications of ×400.

### Statistical Analysis

The results obtained were collated and expressed as Mean ± Standard Error of Mean (S.E.M) and subjected to one-way analysis of variance (ANOVA). The data were further subjected to a post hoc test using Student Neumann Keuls’ method, and differences with probability values of p < 0.05 were considered statistically significant. The statistical analysis was carried out with the aid of GraphPad Prism 5.03 (GraphPad Software Inc., CA, USA).

## Results

### Percentage Yield of PEVA

The result (Table 2) showed that the percentage yield of PEVA is 8.28 ± 0.15 (n=3).

**Table 2:** Percentage (%) Yield of PEVA

	Weight of air-dried and pulverized leaves (g)	Yield of PEVA (g)	Percentage (%) yield of PEVA
1st Extraction Process	500	41.25	8.25
2nd Extraction Process	500	40.20	8.04
3rd Extraction Process	500	42.80	8.56

### Food Consumption (g) and Body Weight (g)

**Figure 1:**
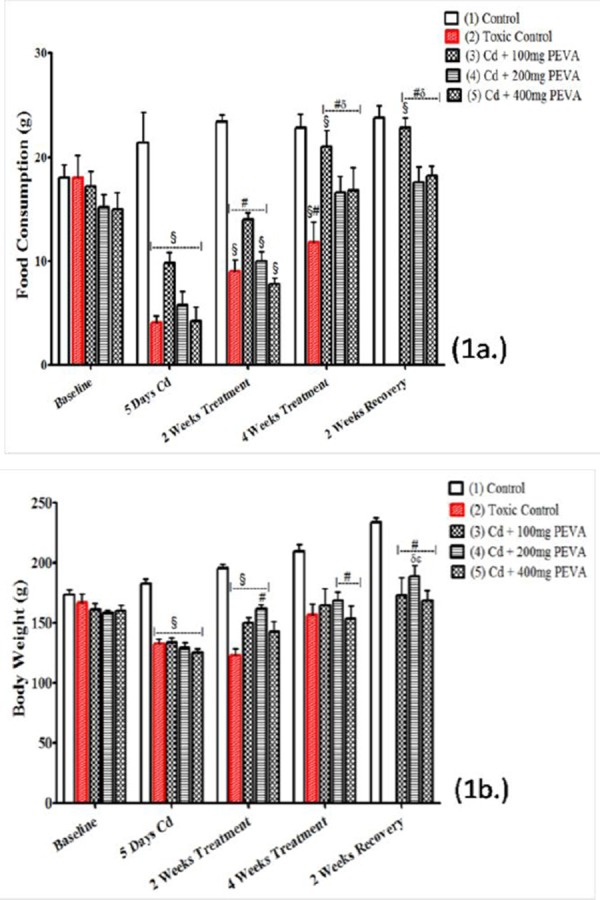
*Effects of PEVA on food consumption (a.) and body weight (b.) of rats exposed to cadmium toxicity*.

The study recorded a significant decrease in the food consumption of rats (Figure 1a) in all Cd-treated groups, that is Groups 2, 3, 4 and 5 (4.10 ± 0.64, 9.80 ± 1.07, 5.80 ± 1.28 and 4.20 ± 1.36, respectively) when compared with their respective baseline (18.00 ± 2.18, 17.20 ± 1.38, 15.20 ± 1.19 and 15.00 ± 1.61) (p < 0.05). At the fourth week of the study, a significant increase (86.39 ± 14.89 %) was observed in these groups when compared with the 2 weeks PEVA-treated groups (14.00 ± 0.63, 10.00 ± 0.89 and 7.80 ± 0.58, respectively) (p < 0.05). However, no significant decrease was recorded in Group 2 (11.80 ± 1.93) at the end of the study when compared with the baseline (18.00±2.18) (t = 2.129; F = 1.276; P = 0.0659).

There was a significant decrease in the body weight of rats (Figure 1b) in all Cd-treated groups, that is Groups 2, 3, 4 and 5 (132.6 ± 7.11, 133.6 ± 4.32, 129.6 ± 4.27 and 125.2 ± 3.43) when compared with their respective baseline (167.0 ± 7.29, 161.4 ± 5.20, 158.8 ± 2.33 and 160.4 ± 4.45) (p < 0.05). At the end of the study, the toxic group (2) never recovered from the weight loss associated with exposure to Cd toxicity (167.0±7.29 to 157.0±9.11). However, a statistically insignificant increase in body weight of the PEVA-treated groups, that is Groups 3, 4 and 5 (173.0 ± 15.05, 189.2 ± 8.53 and 168.6 ± 8.80, respectively) was recorded when compared with their respective baselines (161.4 ± 5.20, 158.8 ± 2.33 and 160.4 ± 4.45) (p > 0.05) at the end of the study period.

### Relative Kidney Weight (%)

**Figure 2:**
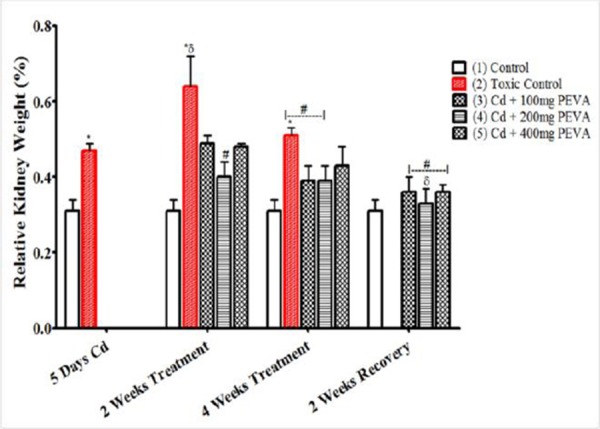
Effects of PEVA on the relative kidney weight of rats exposed to cadmium toxicity. Each value represents mean ± S.E.M (n = 5); *=significantly different from control (p < 0.05); δ = significantly different from toxic group (p < 0.05); #= significantly different from 2 weeks Cd + Recovery group (p < 0.05).

The study recorded a significant increase in relative kidney weight of rats (Figure 2) in Group 2 (0.42 ± 0.02) when compared with Group 1 (0.31 ± 0.03) (t = 3.051; F = 2.250; p < 0.05). The PEVA-treated groups (3, 4 and 5) recorded no significant difference at week two (0.49 ± 0.02; 0.40 ± 0.04; and 0.48±0.01, respectively) when compared with group 2 (0.47 ± 0.02). There was a duration dependent significant decrease in Group 2 between weeks 2 and 4 of recovery period (0.64 ± 0.08 and 0.41 ± 0.02, respectively).

### Plasma Creatinine (mg/dl), Urine Creatinine (mg/dl) and Creatinine Clearance (ml/min ×10^-3^)

**Figure 3:**
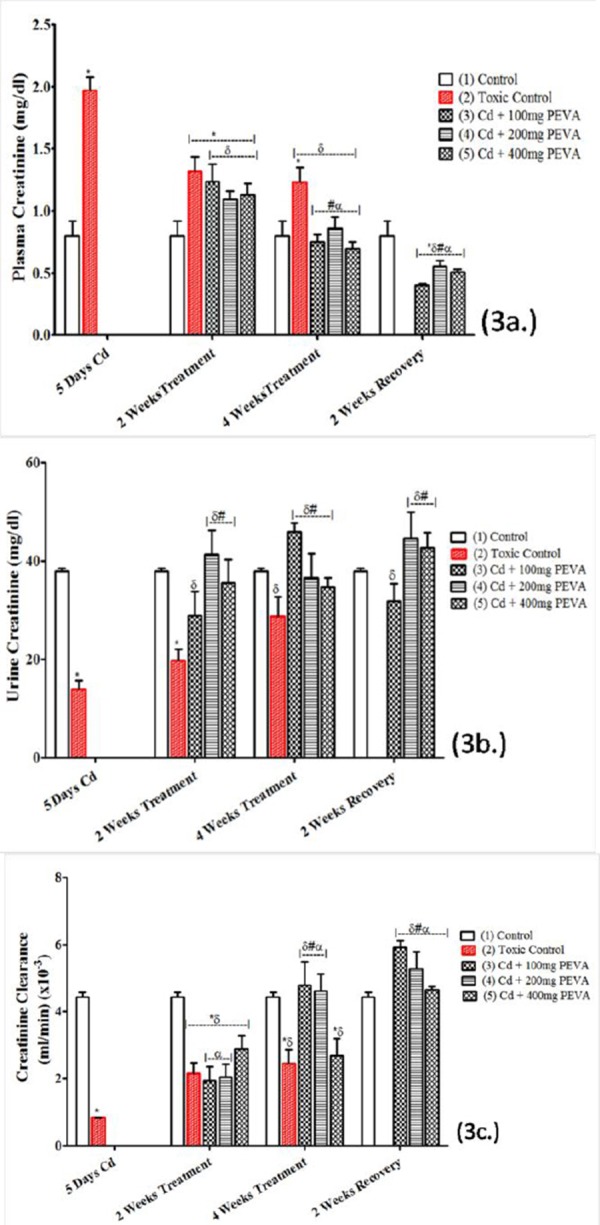
Effects of PEVA on plasma creatinine (a.), urine creatinine (b.) and creatinine clearance (c) of rats with Cd-induced kidney injury. Each value represents mean ± S.E.M (n = 5); *= significantly different from control (p < 0.05); δ= significantly different from toxic group (p < 0.05); #= significantly different from 2 weeks toxic recovery group (p < 0.05); α= significantly different from 4 weeks toxic recovery group (p < 0.05).

Exposure to Cd toxicity induced a significant increase in plasma creatinine of the rats (Figure 3a) in the Group 2 (1.97 ± 0.01) when compared with Group 1 (0.80 ± 0.02) (t = 52.32; F = 4.000; p < 0.05). This was accompanied by a significant decrease in urine creatinine levels (Figure 3b) in the Group 2 (13.86 ± 1.85) when compared with Group 1 (37.84 ± 0.71) (t = 52.32; F = 6.789; p < 0.05).

Also, the period of Cd intoxication was marked with a significant decrease in the creatinine clearance of the rats (Figure 3c) in Group 2 (0.84 ± 0.005) when compared with Group 1 (4.43 ± 0.14) (t = 25.63; F = 784.0; p < 0.05). These alterations were, however, observed to be attenuated in duration, non dose-dependent fashion when compared with the respective control values (Figure 3a, b and c).

### Plasma Urea (mg/l), Urine Urea (g/l) and Urea Clearance (ml/min ×10^-5^)

**Figure 4:**
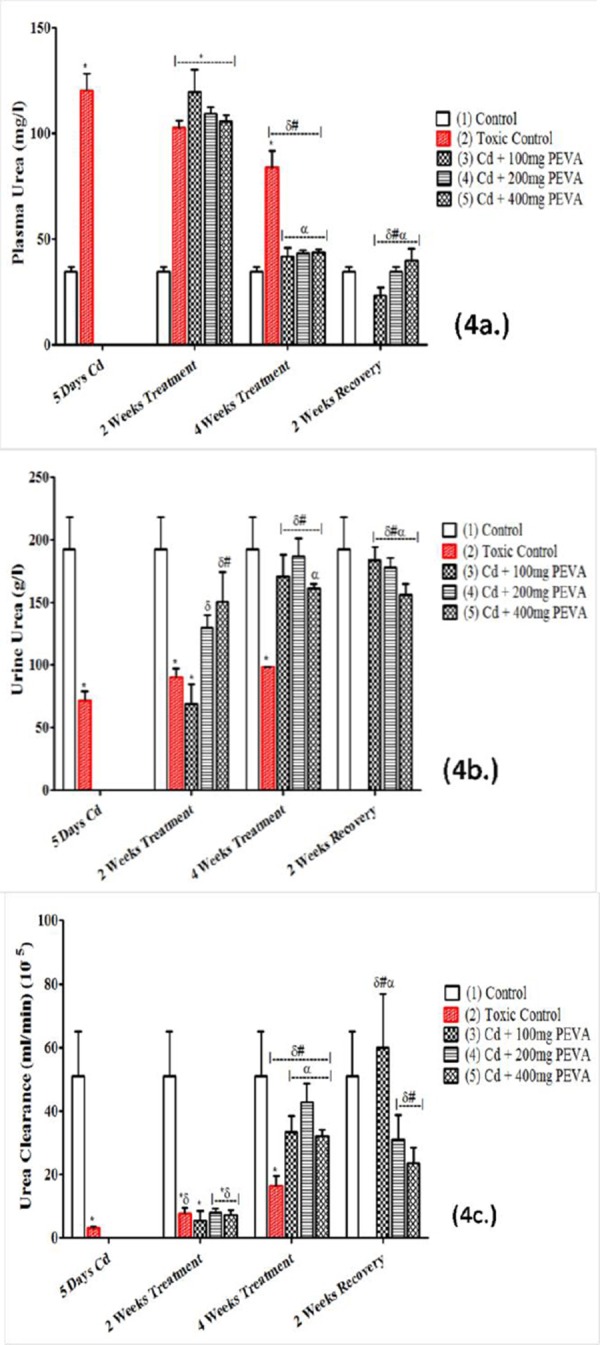
Effects of PEVA on plasma Urea (a.), urine urea (b.) and urea clearance (c) of rats with Cd-induced kidney injury. Each value represents mean ± S.E.M (n = 5); *= significantly different from control (p < 0.05); δ= significantly different from toxic group (p < 0.05); #= significantly different from 2 weeks toxic recovery group (p < 0.05); α= significantly different from 4 weeks toxic recovery group (p < 0.05).

Cd intoxication induced a significant increase in plasma urea of the rats (Figure 4a) in Group 2 (120.60 ± 7.85) when compared with Group 1 (34.69 ± 2.32) (t = 10.50; F = 11.45; p < 0.05). This was accompanied by a significant decrease in urine urea of the rats (Figure 4b) in Group 2 (192.50 ± 0.52) when compared with Group 1 (71.14 ± 0.80) (t = 127.2; F = 2.367; p < 0.05). Also recorded was a significant decrease in the urea clearance of rats in Group 2 (3.17 ± 0.62) when compared with Group 1 (51.00 ± 14.00) (t = 3.41; F = 509.90; p < 0.05), during the period of Cd intoxication. These Cd-induced alterations in urea levels were also observed to be reversed in duration, non dose-dependent fashion when compared with the respective control values (Figure 4a, b and c).

### Plasma Glucose (mg/dl) and Urine Glucose (mg/ml)

**Figure 5:**
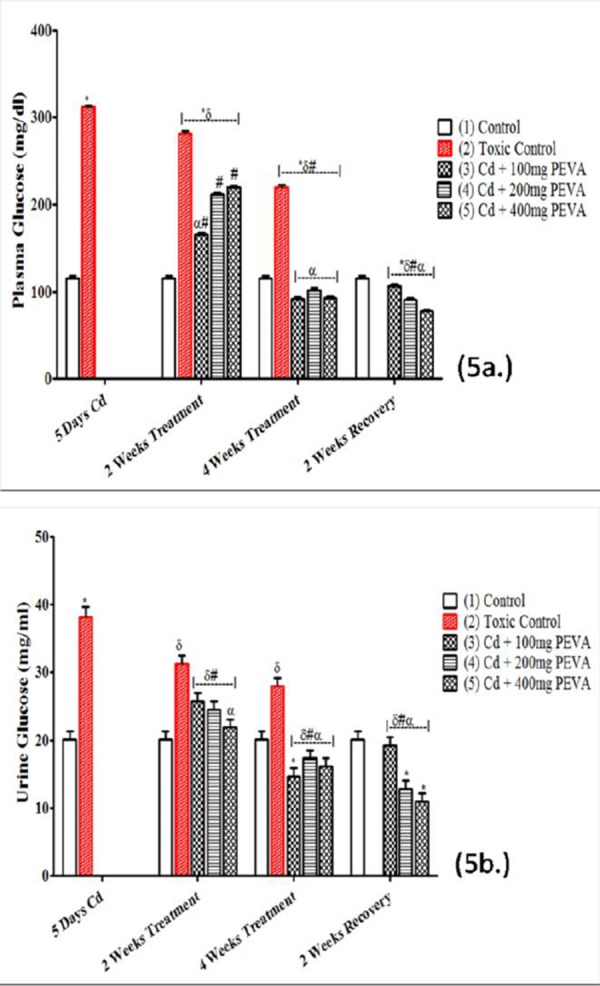
Effects of PEVA on plasma glucose (a.) and urine glucose (b.) of rats with Cd-induced kidney injury. Each value represents mean ± S.E.M (n = 5); *= significantly different from control (p < 0.05); δ= significantly different from toxic group (p < 0.05); #= significantly different from 2 weeks toxic recovery group (p < 0.05); α= significantly different from 4 weeks toxic recovery group (p < 0.05).

Both plasma glucose (311.87 ± 1.36) (Figure 5a) and urine glucose levels (38.19 ± 1.39) (Figure 5b) were found to be significantly increased in the Group 2 when compared with their respective Group 1; (115.31 ± 1.86) (t = 85.31; F = 1.870; p < 0.05) and (20.10 ± 1.21) (F = 1.320; p < 0.05). PEVA treatment attenuated plasma and urine glucose aberrations in rats exposed to Cd toxicity in a dose-dependent fashion with a relative potency of 400 mg/kg > 200 mg/kg > 100 mg/kg (Figure 3.7a & b). However, a significant decrease in plasma glucose was recorded in Group 2 at 2 week recovery period (281.78 ± 2.40) when compared with the toxic group (311.87 ± 1.36) (F= 3.114; p<0.05).

This was found to be significantly decreased in the PEVA-treated groups (3, 4 and 5) at both week 2 (165.20 ± 1.82; 211.61 ± 1.79; and 220.02 ± 1.56, respectively) and week 4 (92.10 ± 2.02; 101.97 ± 2.08; and 92.28 ± 2.09, respectively). At the end of the study, there was a significant decrease in the urine level of glucose in the PEVA-treated groups (3, 4 and 5) (14.62 ± 1.21; 17.36 ± 1.14; 16.08 ± 1.25) when compared with Group 2 (38.19±1.39) (F = 79.34; p < 0.0001).

### Non- enzymatic Antioxidant Status (GSH) (μg/mg protein) and Levels of Lipid Peroxidation (TBARS) (nmol/mg protein)

**Figure 6:**
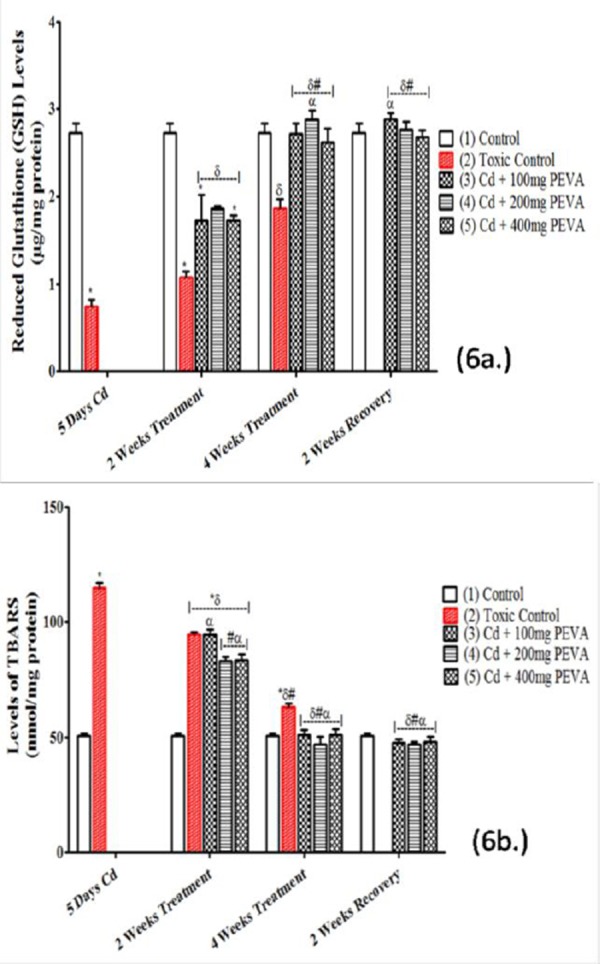
Effects of PEVA on renal levels of GSH (a.) and TBARS (b.) in rats with Cd-induced kidney injury. Each value represents mean ± S.E.M (n = 5); *= significantly different from control (p < 0.05); δ= significantly different from toxic group (p < 0.05); #= significantly different from 2 weeks toxic recovery group (p < 0.05); α= significantly different from 4 weeks toxic recovery group (p < 0.05).

The GSH level in the renal tissue of the rats (Figure 6a) in Group 2 (0.74 ± 0.08) was significantly decreased when compared with Group 1 (2.73 ± 0.11) (t = 14.63; F = 1.891; p < 0.05). There was no significant difference in the PEVA-treated groups (3, 4 and 5) at 2 weeks recovery period (2.88 ± 0.07; 2.77 ± 0.09; and 2.68 ± 0.08, respectively) when compared with 4 weeks PEVA-treated groups (2.72 ± 0.12; 2.88 ± 0.10; and 2.62 ± 0.16, respectively) and Group 1 (2.73 ± 0.11).

Also recorded was a statistically significant decrease in during 2 weeks recovery period in Group 2 (1.07 ± 0.07) when compared with 4 weeks recovery period in Group 2 (1.86 ± 0.23) (F = 2.469; p < 0.05).

There was a significant increase in TBARS level (Figure 6b) in the Group 2 (114.90 ± 2.16) when compared with the Group 1 (50.63 ± 1.32) (t = 25.39; F = 2.678; p < 0.05). There was no significant difference in the PEVA-treated groups (3, 4 and 5) at 2 weeks recovery period (47.82 ± 1.44; 46. 97 ± 1.21; 48.23 ± 2.20) when compared with the PEVA-treated groups at week 4 (51.07 ± 2.15; 46.99 ± 3.21; 51.23 ± 2.33) as well as with Group 1 (50.63 ± 1.32). Also recorded was a statistically significant decrease in levels of TBARS in Group 2 at 2 weeks recovery (94.54 ± 1.27) when compared with 4 weeks recovery period (63.23 ± 1.31) (F= 1.064; p < 0.05).

### Histopathological Investigation

**Figure 7:**
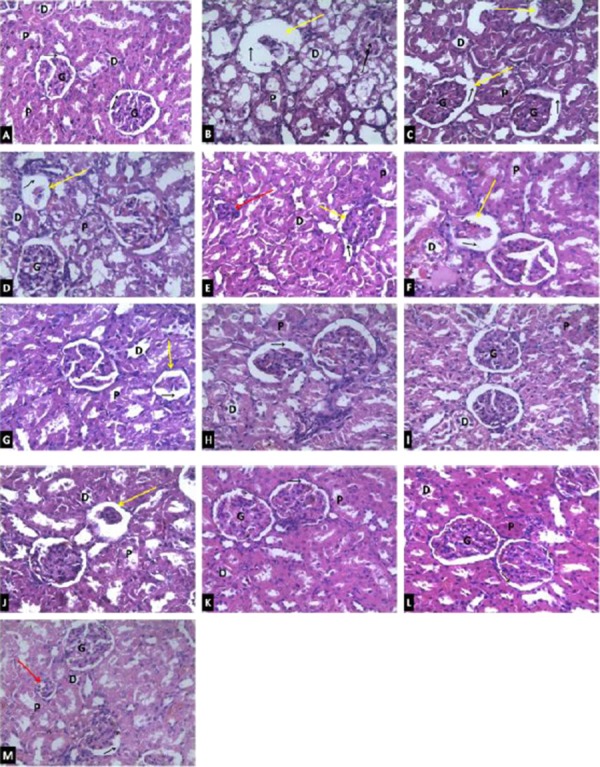
*Photomicrographs of renal tissues showing the effects of PEVA treatment following Cd-induced kidney injury in rats (Magnification 400x). A = Control group; B = Toxic group (5 days Cd treatment); C = Toxic group (Cd + 2 weeks recovery period); D = Toxic group (Cd + 4 weeks recovery period); E = Cd + 100 mg/kg PEVA for 2 weeks; F = Cd + 200 mg/kg PEVA for 2 weeks; G = Cd + 400 mg/kg PEVA for 2 weeks; H = Cd + 100 mg/kg PEVA for 4 weeks; I = Cd + 200 mg/kg PEVA for 4 weeks; J = Cd + 400 mg/kg PEVA for 4 weeks; K = 100 mg/kg PEVA treatment + 2 weeks recovery period; L = 200 mg/kg PEVA treatment + 2 weeks recovery period; M = 400 mg/kg PEVA treatment + 2 weeks recovery period; Yellow arrow = atrophic glomerulus; Red arrow = shrunken glomerulus; Black single arrow = Bowman’s capsular space; Black double arrow = Severe cloudy swelling of the proximal convoluted tubule; G = Glomerulus; P = Proximal convoluted tubules and D = Distal convoluted tubules*.

The photomicrograph of the toxic group (Figure 7B) showed evidence of distorted renal corpuscles with atrophic glomerulus (yellow arrow), loss of cellular constituents in the tubules and severe cloudy swelling of the proximal convoluted tubule (black double arrow). This was in contrast to the control group (Figure 7A) that showed intact renal corpuscles with normal appearing glomerulus (G) and tubules; proximal convoluted tubules (P), distal convoluted tubules (D) as well as intact Bowman’s capsular space (black single arrow).

The Cd + recovery groups (Figures C and D) showed a duration-dependent improvement in the renal histoarchitecture of the rats when compared with the toxic group. However, these improvements were found to be less significant in a duration-dependent fashion when compared with the PEVA-treated groups at 2 weeks of PEVA treatment (Figures 7 E, F and G), 4 weeks of PEVA treatment (Figures 7 H, I and J) and with the groups that were left to recover for 2 weeks after PEVA treatment (Figures 7 K, L and M). Within the PEVA-treated groups, the improvements that were observed were duration-dependent but not dose-dependent. This was with a relative potency of 200 mg/kg > 100 mg/kg > 400 mg/kg body weight of PEVA administration. It is worthy of note to state that the groups that received 400 mg/kg PEVA still showed marked evidence of atrophic (yellow arrow) and shrunken (red arrow) glomeruli even after 4 weeks of PEVA treatment (Figure 7J) and 2 weeks recovery period after PEVA treatment (Figure 7M).

## Discussion

The study was in three phases. The first and second phases investigated the effects of two and four weeks treatment with polyphenol-rich extract of leaves of *Vernonia amygdalina* (PEVA), respectively, on the renal function of rats with cadmium-induced kidney injury while the third phase investigated the impact of a two week recovery period from PEVA treatment in rats with cadmium-induced kidney injury.

Exposure to Cd toxicity was associated with tiredness and reduced appetite, as observed in the rats. This explains the reduction in body weight that was recorded in the study. Katherine *et al* [23] reported that in any individual, weight gain or loss is a function of a balance between food consumption and the rate of energy expenditure. Therefore, the reduction in body weight that was recorded was a consequence of Cd-induced alteration in feeding pattern. Within neural circuitry, the “key controller” that maintains energy homeostasis is the hypothalamus [23]. The brain has been reported in literature to also be an area of Cd bioaccumulation [24, 25, 26] with undesirable effects on the hypothalamus [27, 28]. Subject to further investigation and verification, Cd toxicity may have resulted in the suppression of the hypothalamic centre for energy expenditure and food consumption, with a consequent reduction in appetite. PEVA treatment was associated with a duration-dependent improvement in the appetite and feeding pattern of rats, suggesting a possible modulatory effect of PEVA on appetite to restore homeostasis of feeding pattern in subjects that have been exposed to Cd toxicity.

The physiological explanation for the increase in both plasma and urine glucose levels is *splay*. This is the concentration difference between a substance transport maximum and its renal threshold. It is evident that the splay for blood glucose was significantly reduced during the period of exposure to Cd toxicity; since the magnitude of splay is inversely proportional to the avidity with which the transport mechanism binds the substance it transports [29]. Cd has been reported to target various transporters in brush borders and baso-lateral membranes of the kidney [30]. This study recorded that Cd toxicity induced hyperglycemia which was sufficient to induce glycosuria, with the renal threshold for glucose being exceeded. However, the representative photomicro-graph of the toxic group revealed loss of cellular constituents and severe cloudy swelling in the proximal convoluted tubules, where almost all glucose reabsorption takes place [31]. This suggests that the mechanism by which Cd induced glycosuria was both induction of hyperglycemia and reduction in renal reabsorptive capacity for glucose. PEVA treatment was observed to have significantly reversed the Cd-induced alteration in plasma and urine levels of glucose in a dose-dependent fashion, suggesting its potential in restoring body glucose homeostasis. It is, however, worthy of note to state that the dose-dependent decrease in the plasma glucose level that was associated with PEVA treatment culminated in hypoglycemia in the group that received the highest dose, in a manner that was duration-dependent. This suggests a high risk profile of PEVA administration at high doses.

The significant increase in the relative kidney weight (RKW) that was recorded is in contrast to some reports on the effects of Cd toxicity on RKW. For instance, Borde *et al* [32] and Onwuka *et al* [33] reported that Cd toxicity induced significant decrease in RKW. These contrasting reports could have been as a result of difference in the salts of Cd that were used for the studies; while this study investigated Cd in the form of cadmium sulphate, the former investigated cadmium in the form of cadmium chloride. Similar finding (significant increase in RKW) was reported by Ige *et al* [34], who used cadmium in form of cadmium sulphate. This could mean that salts of Cd can induce toxicity in unique patterns that are peculiar to each salt. The significant increase may have resulted from the features of severe cloudy swelling as revealed in the representative photomicrograph. An apparent anti-inflammatory potential of PEVA was demonstrated by its ability to significantly reverse the Cd-induced increase in RKW.

The increased level of TBARS observed in the renal tissues of rats with Cd toxicity indicated a high degree of lipid peroxidation and oxidative stress. Lipid peroxidation, though indirectly involved in the generation of free radicals, is considered as the primary mechanism for Cd-induced toxicity [35-38]. Because of the presence of sulfhydryl group in it, GSH is a non-enzymatic antioxidant which acts as a first line of defense against oxidative stress [39]. The significant reduction in GSH levels that was recorded can be attributed to the increased use of GSH (by the renal tissues) to mop up excessive Reactive Oxygen Species (ROS) that were generated during the process of Cd-induced renal injury. Inducing ROS generation through oxidative damage to cellular organelles is the mechanism by which Cd exerts its toxic effects [40]. Reactions of these ROS, which consist mainly of O_2_^+^, H_2_O_2_ and OH^+^ [41], with cellular biomolecules have been shown to lead to lipid peroxidation, membrane protein damage, altered anti-oxidant system, altered gene expression, DNA damage and apoptosis [42, 40]. The GSH report in this study is consistent with the other reports in which GSH concentration was decreased during exposure to Cd toxicity [43, 41]. The ability of Cd to generate free radicals is a pointer to the possibility of ameliorating the oxidative stress that it induces with the use of potent antioxidants. PEVA administration to rats that were exposed to Cd toxicity reinstated normal levels of both TBARS and GSH in the renal tissues, possibly by significantly reducing Cd reaction with cellular biomolecules. This suggests that PEVA is a potent antioxidant. The efficacy of PEVA in this study, as an antioxidant, became obvious during the 4th week treatment period and therefore sustained.

The protection of cell constituents against oxidative damage through scavenging of free radicals is a concept that is considered an oversimplified view of the mechanism of polyphenol action [44]. The biological effects of polyphenols may far exceed the modulation of oxidative stress in biological systems [45].

The record on plasma and urine creatinine level is consistent with previous findings [46, 47, 48, 49]. A significant increase in plasma levels of creatinine is usually associated with a compromise to the filtering capacity of the kidney [50] hence, creatinine clearance is a function of glomerular filtration rate. As depicted by features of atrophic glomeruli, the significant increase in plasma creatinine can be attributed to a compromise to the renal filtration capacity following exposure to Cd toxicity. Also, after a significant decrease, creatinine clearance was observed to be significantly increased in a duration-dependent fashion in the PEVA-treated groups. This potential of PEVA to significantly reverse the Cd-induced alteration in creatinine clearance, buttressed by the corresponding photomicrographs, may have resulted from its antioxidant effect. However, the observations of this study portray PEVA as having a potential role, possibly, in tissue regeneration as a means of improving levels of plasma creatinine as well as creatinine clearance in subject exposed to Cd toxicity. The (precise) mechanism by which polyphenols reinstate normal levels of creatinine in biological systems is worthy of further investigation.

The integration of amino acids into proteins is inhibited by Cd, causing an increase in blood urea level [49]. Also, tubular secretion and reabsorption (through urea transport proteins) are the major factors that regulate the excretion of urea from the body [51]. PEVA’s ability to significantly reinstate normal levels of urea suggests its potential to enhance the integration of amino acids into proteins, thus abating Cd-induced nephritis and or mediate possible alterations in urea transport proteins in the renal tubules to restore urea homeostasis. The representative photomicrographs depicted intact renal histoarchitecture when compared with that of the control. This is, however, subject to further investigation and verification.

It is noteworthy to state that rats that were left untreated after exposure to Cd toxicity showed remarkably steady degree of amelioration when compared with the toxic group. However, when compared with the PEVA-treated groups, there was a significant difference; with PEVA-treated groups showing between “two-fold to over-six fold” increase in ameliorative capacity as opposed to when rats were left untreated. Based on this observation, it can be deduced that renal tissues of rats with Cd-induced kidney injury have the potential to attempt the restoration of normal renal function in a duration-dependent manner, provided complete abstinence from Cd exposure is maintained.

Although PEVA showed significant changes in the biochemical and histological aberrations that were induced by exposure to Cd toxicity, its relative potency was in the order 200 mg/kg PEVA> 100 mg/kg PEVA> 400 mg/kg PEVA. This shows that the ameliorative potential of the extract is not dose-dependent. Also, prolonged administration of high doses can potentiate further injury rather than sustaining ameliorative potentials. It was observed that a 2 week administration of PEVA for the adopted graded doses in the PEVA-treated groups produced effects that were similar to the toxic recovery groups for all biochemical parameters that were measured. This fact was supported by the representative photomicrographs, thus indicating that the beneficial effects of PEVA administration are duration-dependent. Therefore, there must be a balance between choice of therapeutic dose and duration of administration.

It is recommended that the consumption of PEVA or, at least, fresh leaves (rather than blanching) of *Vernonia amygdalina* should be encouraged in order to maximize its health benefits as a relatively cheaper and potential herbal alternative in the treatment or management of subjects with Cd-induced kidney injury, which is otherwise expensive to manage or treat with the use of conventional therapy.****

In conclusion, the administration of polyphenol-rich extract of the leaves of Vernonia amygdalina (PEVA) reversed the compromise of renal function that was induced by cadmium toxicity in rat model. The antioxidant, anti-inflammatory and membrane stabilizing activities can be considered as the key factors responsible for the ameliorative effect of PEVA. Therefore, it represents a prospective therapeutic choice to ameliorate the renal oxidative injury inflicted by Cd in exposed subjects. Nevertheless, a high risk profile at high doses is not unlikely.
